# Versican contributes to ligament formation of knee joints

**DOI:** 10.1371/journal.pone.0250366

**Published:** 2021-04-22

**Authors:** Tomoko Higuchi, Daisuke Suzuki, Takafumi Watanabe, Kanda Fanhchaksai, Keiko Ota, Kazuhisa Yokoo, Hiroshi Furukawa, Hideto Watanabe

**Affiliations:** 1 Department of Plastic Surgery, Aichi Medical University, Nagakute, Japan; 2 Department of Health Sciences, Hokkaido Chitose College of Rehabilitation, Chitose, Japan; 3 Laboratory of Veterinary Anatomy, School of Veterinary Medicine, Rakuno Gakuen University, Ebetsu, Japan; 4 Institute for Molecular Science of Medicine, Aichi Medical University, Nagakute, Japan; University of Bologna, ITALY

## Abstract

Versican is a large proteoglycan in the extracellular matrix. During embryonic stages, it plays a crucial role in the development of cartilage, heart, and dermis. Previously, we reported that *Prx1-Vcan* conditional knockout mice, lacking Vcan expression in mesenchymal condensation areas of the limb bud, show the impaired joint formation and delayed cartilage development. Here, we investigated their phenotype in adults and found that they develop swelling of the knee joint. Histologically, their newborn joint exhibited impaired formation of both anterior and posterior cruciate ligaments. Immunostaining revealed a decrease in scleraxis-positive cells in both articular cartilage and ligament of *Prx1-Vcan* knee joint, spotty patterns of type I collagen, and the presence of type II collagen concomitant with the absence of versican expression. These results suggest that versican expression during the perinatal period is required for cruciate ligaments’ formation and that its depletion affects joint function in later ages.

## Introduction

The joint is formed by apoptosis of undifferentiated mesenchymal cells in the cartilage primordium of long bones. Mesenchymal cells remain pre-differentiated chondrocytes and go through apoptosis, where hyaluronan (HA) is accumulated, forming a cavity named joint interzone. The marginal cells retain pre-chondrocytic nature and serve as articular chondrocytes. In this process, versican (Vcan) [1], a chondroitin sulfate (CS) proteoglycan of the extracellular matrix (ECM) type, is expressed with dynamic patterns. Its expression initiates in mesenchymal condensation areas. During the differentiation of mesenchymal cells into chondrocytes, it remains in pericondensation areas. When the joint formation occurs through the accumulation and lining up of mesenchymal cells, Vcan expression is restricted to the joint interzone. After the joint cavity formation, Vcan is localized in the articular cartilage and synovial tissue, lining the inside margin of the cavity [2, 3].

Vcan contains a core protein consisting of an N-terminal G1, CSα, CSβ, and C-terminal G3 domains. The N-terminal G1 domain consists of A, B, and B’ subdomains and binds to both HA and link protein (LP) [4]. Both CSα and CSβ domains are attached with CS chains. The C-terminal G3 domain binds different ECM molecules such as fibrillin-1 [5], fibulin-1 and -2 [6, 7], tenascins [6, 8], and heparan sulfate proteoglycans [9]. Vcan exhibits four spliced variants; V0 (G1-CSα-CSβ-G3), V1 (G1-CSβ-G3), V2(G1-CSα-G3), and V3(G1-G3) [10–13]. Whereas V0 and V1 are widely expressed, V2 expression is restricted to the nervous systems. Thus, the number of CS chains necessary for the function of Vcan may vary among tissues.

Previous cell culture studies have revealed various effects of Vcan on cell behavior [14]. For example, Vcan inhibits the adhesion of osteosarcoma cells and aggravates their malignant phenotype [15]. It inhibits neural crest cell migration and the outgrowth of motor and sensory axons [16]. The function of Vcan appears to be through the G1 domain regulating HA-mediated signaling, the G3 domain mediating TGFβ- and BMP-signaling, CS chains binding cytokines and growth factors such as midkine and pleiotrophin, and epidermal growth factor (EGF)-like motifs in the G3 domain binding to EGF receptors [14].

In addition to developing cartilage and forming joints, transient Vcan expression is observed in various developing tissues [17], including the brain [18], developing heart [19, 20], hair follicles [21, 22], and tooth and periodontal tissues during tooth eruption [23]. In the brain, Vcan promotes presynaptic maturation [24]. Vcan contributes to cardiac jelly formation and septal formation [25, 26]. Vcan in dense aggregates of dermis-derived stromal cells suggests its involvement in hair follicle formation [21]. These studies suggest that Vcan plays the central role in the formation of a provisional matrix [1, 27].

Previously, we generated and analyzed *Prx1*-Cre/*Vcan*^flox/flox^ (herein *Prx1-Vcan*) mice, which lack Vcan expression in mesenchymal condensation areas of cartilage primordium. Whereas these mice are viable and fertile, they exhibit delayed chondrocyte differentiation and joint malformation in digits [28]. Further analysis revealed decreased TGFβ accumulation and down-regulation of its signaling required for chondrocyte differentiation, indicating that Vcan is necessary for the local concentration of TGFβ and its adequate signaling.

While maintaining *Prx1-Vcan* mice, we found the laxity of knee joints, which can be observed as early as six months. As Vcan is expressed in tendons and ligaments under physiological conditions [29, 30], and in tendinopathy [31, 32], lack of Vcan expression in tendons and ligaments may cause joint destruction. Here, we investigated abnormalities in their joint and found malformation of cruciate ligaments in newborn mice. These results suggest that Vcan expression in the perinatal period is necessary for the normal formation of jointed appendages and that its impairment causes joint laxity later.

## Experimental procedures

### Mice

All the experiments were approved by the animal care committee at Aichi Medical University. *Prx1*-Cre*/Vcan*^flox/flox^ (*Prx1-Vcan*) and *Prx1*-Cre */Vcan*^+/+^(*Prx1-Vcan*^*+/+*^) mice were maintained, and their genotyping was performed as reported previously [28]. *Prx1-Vcan* and *Prx1-Vcan*^*+/+*^ mice were crossed several times with *mT/mG* reporter mice, and *Prx1-Vcan*: *mT/mG* and *Prx1-Vcan*^*+/+*^: *mT/mG* mice were generated and maintained under physiological conditions. Usually, two~three mice were in a cage.

### Histology and immunohistochemistry

Mice were sacrificed at four~six months. Their limbs were fixed in 10% buffered formalin for 24 h and decalcified using KCX (Falma, Tokyo) overnight. Newborn mice were sacrificed, and their limbs were fixed in 4% paraformaldehyde/phosphate-buffered saline (PBS) for 24 h. After embedding the samples in paraffin, deparaffinized sections (3–5 μm) were prepared for hematoxylin and eosin (H&E) and immunostaining. Step-wise section slides of every five slices and serial sections for four~six-month-old joints and newborn joints were prepared, respectively. The primary antibodies used were as follows: rabbit polyclonal anti-Vcan GAGβ(×500 dilutions; Millipore), goat anti-enhanced green fluorescent protein (EGFP, ×200; Rockland), anti-type I collagen (×200; LSL, LB-1102), and anti-type II collagen (x200; LSL, LB-1297). For secondary antibodies, anti-rabbit or anti-goat IgG conjugated with Alexa594 (Molecular Probes) and Alexa488 (Molecular Probes) were used. For observation, the BZ 9000 (Keyence) was used as light and immunofluorescence microscopy.

### microCT analysis

Limbs at five months were scanned for baseline microarchitecture in a randomized sequence, using a CosmoScan RmCT2 microCT machine (Rigaku, Tokyo). The joint region, including femur, tibia, and fibula, was evaluated.

### FACS analysis

Skin was collected from *Prx1; mT/mG* newborn hindlimbs, washed with PBS ten times, treated with 0.05% trypsin-EDTA overnight at 4 °C, and then with 0.35% collagenase type I (Roche) for 2 h. The sample was centrifuged at 1,000 ×g for 5 min. Cells were resuspended in Dulbecco’s modified Eagle’s medium containing 10% fetal bovine serum and analyzed using FACS Canto II^™^ (BD Biosciences).

## Results

**Fig 1:**
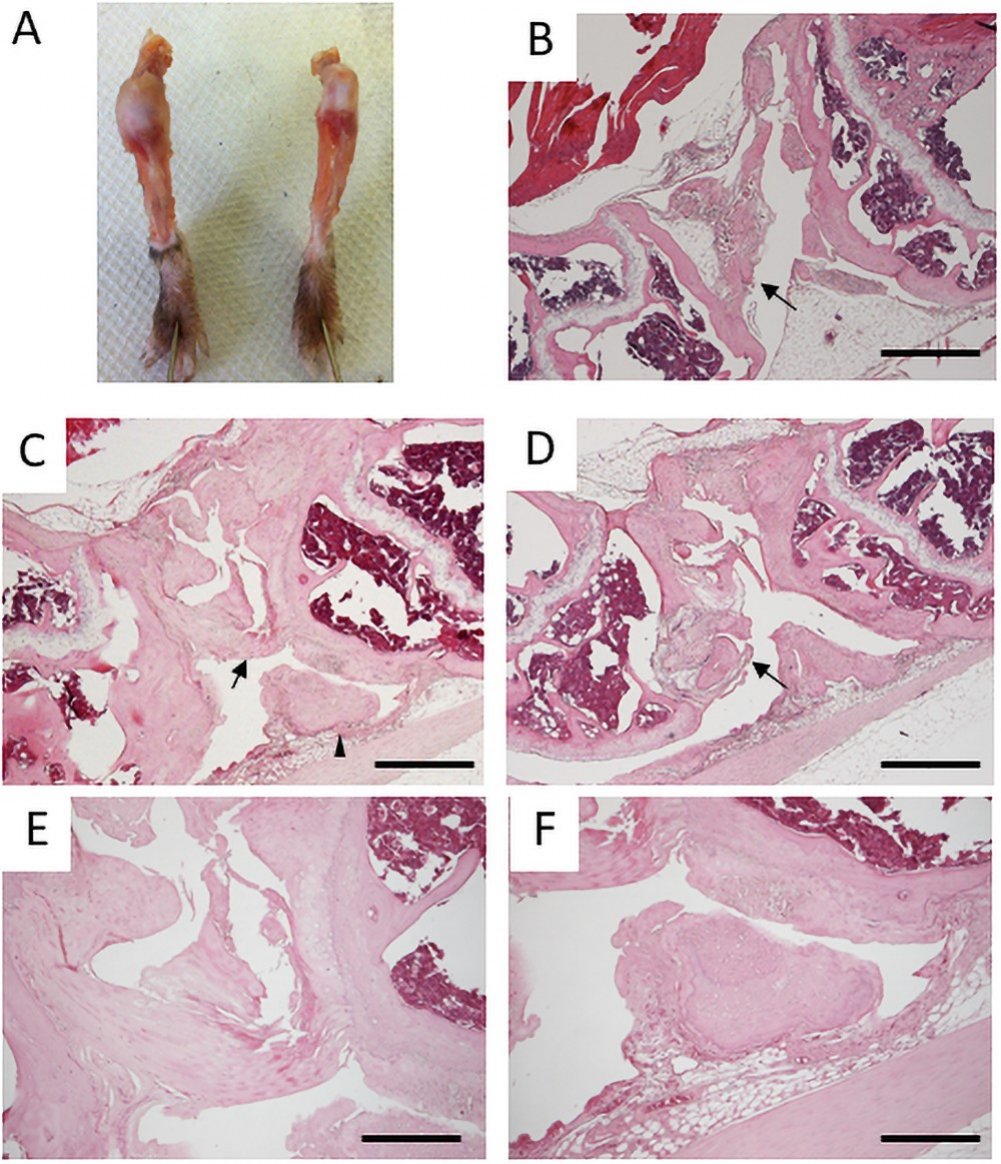
Knee joints of six-month-old mice. A. Gross appearances of knee joint capsules of *Prx1-Vcan* (left) and *Prx1-Vcan*^+/+^ (right). B. Histological Analysis of *Prx1-Vcan*^*+/+*^ knee joint (H&E). Note that the PCL is normal (arrow). C. *Prx1-Vcan* knee joint (H&E). PCL is disorganized (arrow), and joint mouse is observed (arrowhead). D. *Prx1-Vcan* knee joint (H&E). A false joint is observed on the distal end of the femur (arrow). E. High power view of the arrowhead region in Panel C (H&E). Note degeneration of the ligament with calcification assessed by the tidemark. F. High power view of the region of the arrowhead in Panel C (H&E). Note the presence of a small free body composed of cartilaginous tissue. Bar = 500 μm in B-D, 200 μm in E, F.

*Prx1*-Cre/*Vcan*^flox/flox^ (*Prx1-Vcan*) mice are viable and fertile, although they exhibit distorted digits in both fore and hindlimbs compared with *Prx1*-Cre/*Vcan*^+/+^ (*Prx1*-*Vcan*^+/+^) as control. We examined whether they have additional abnormalities during their growth or not. By gross appearance, *Prx1-Vcan* mice were slightly smaller than the *Prx1*-*Vcan*^+/+^. Their knee joint capsule was larger than *Prx1*-*Vcan*^+/+^ (Fig 1A), although it did not show inflammatory responses. It was discernible as early as ~five months, but not at ~four months. Then, we investigated the individual components of the joint region. By microCT analysis at five months, the bones of *Prx1-Vcan* mice did not show any abnormalities (S1 Fig).

Next, we performed histological analysis of six-month-old mice. Whereas articular cartilage of *Prx1*-*Vcan*^+/+^ remained smooth, that of *Prx1-Vcan* was severely disorganized, with fibrillated surfaces (Fig 1B–1D). Whereas the posterior cruciate ligament (PCL) appeared normal (Fig 1B, arrow) in *Prx1*-*Vcan*^+/+^ joint, the PCL of *Prx1-Vcan* joint was tortuous and fused (Fig 1C, arrow). Besides, a small free body composed of cartilaginous tissue, the so-called “joint mouse,” was observed (Fig 1C, arrowhead, and Fig 1F). Another *Prx1-Vcan* joint exhibited marked tissue degeneration (Fig 1D). The distal femoral region formed a “pseudojoint” (Fig 1D, arrow) presumably due to repeated fracture, and the boundary of bones and connective tissue was unclear (Fig 1D, arrowhead). The apparent anterior cruciate ligament (ACL) was disorganized (Fig 1E). Taken together, *Prx1-Vcan* mice at six months of age showed disorganization of cruciate ligaments and formation of a free body.

**Fig 2:**
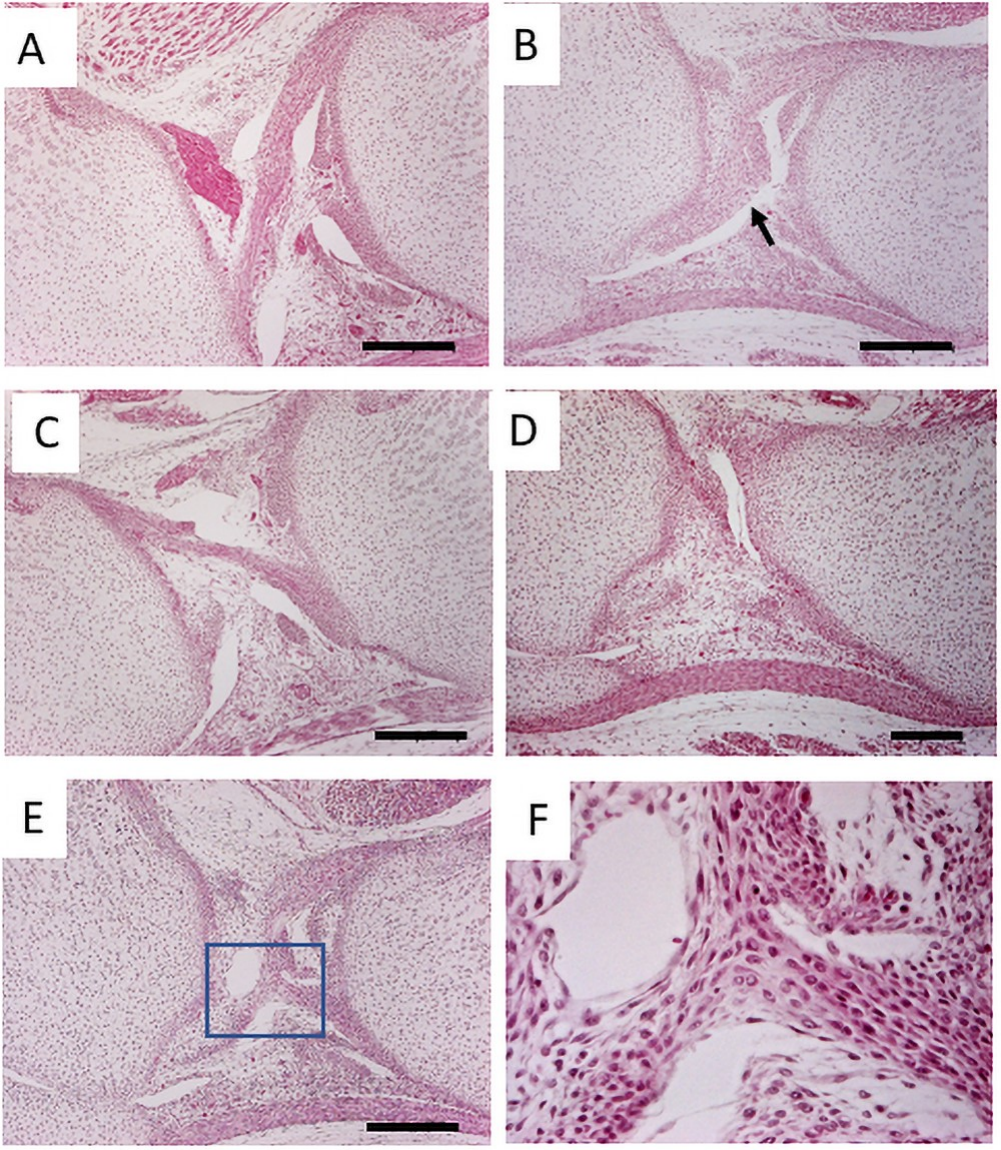
The histological analysis of newborn mice. A, C. The knee joint of *Prx1-Vcan*^+/+^. B. *Prx1-Vcan* joint. Note disorganized ACL (arrow). D. *Prx1-Vcan* joint. Note that the patella fat and anterior femoral region are connected with broader tissues. E. *Prx1-Vcan* joint. ACL and PCL are disorganized and indiscernible (Bar = 200 μm). F. High-power view of the rectangle in E. Cells in the ligament are in different sizes, oriented to different directions.

As Vcan is expressed at high levels in the joint of the perinatal period and rapidly disappears from the joint, we asked whether abnormalities were already initiated in that period. The newborn *Prx1-Vcan* joint exhibited disorganized PCL (Fig 2B, arrow) compared with *Prx1*- *Vcan*^+/+^ joint (Fig 2A and 2C). In *Prx1-Vcan* joint, the patella fat pad and anterior femoral region were connected with broader connective tissues (Fig 2D), whereas *Prx1*- *Vcan*^+/+^ joint barely exhibited connection. In the *Prx1-Vcan* joint, both ACL and PCL were disorganized and indiscernible (Fig 2E), and the ligamental cells showed different sizes and orientations (Fig 2F).

**Fig 3:**
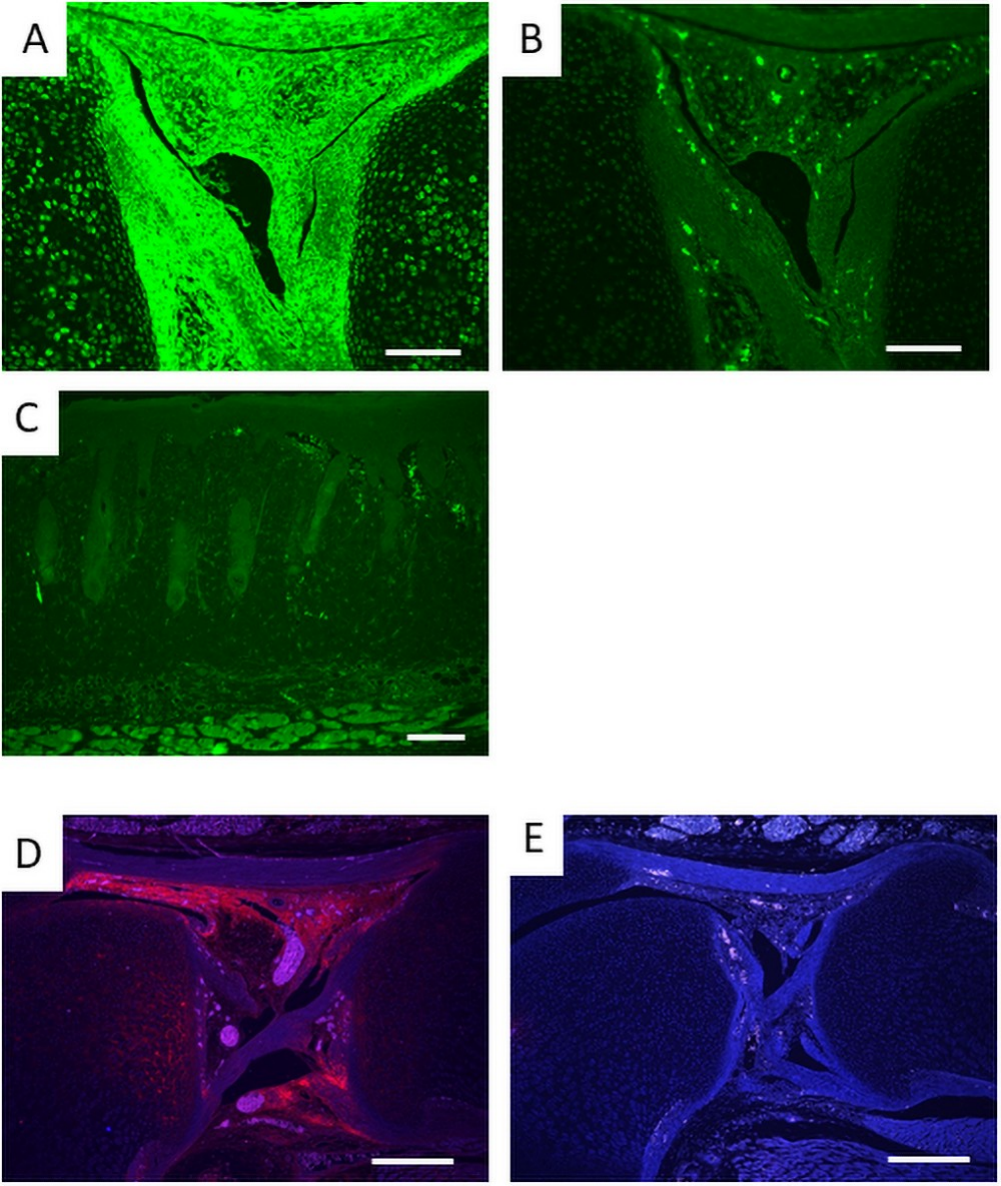
Immunostaining of EGFP and Vcan. A. EGFP of newborn *Prx1-Vcan*: *mT/mG* mouse knee joint. B. Negative control with non-immune IgG. C. EGFP of newborn mouse skin. Note little fluorescence indicating that dermal fibroblasts lack or lacked Prx1 promoter activity. D. Vcan of *Prx1-Vcan*^+/+^ newborn mouse knee joint. E. Vcan of *Prx1-Vcan* knee joint. (Bar = 100 μm in A, B; 200 μm in C, D, E).

In *Prx1-Vcan* mice, Vcan expression is depleted only in the cells with cre promoter activity. To identify them, we performed immunostaining for EGFP on *Prx1*-*mT/mG* mice. EGFP was detected in articular cartilage, synovia, cruciate ligament, and tendon (Fig 3A), but not in the dermis (Fig 3C). When we isolated and cultured dermal fibroblasts from *Prx1-mT/mG mice* and sorted by FACS, no green fluorescent fibroblasts were detected (S2 Fig), indicating that *Prx1* promoter was and had been inactive in dermal fibroblasts. By immunostaining, Vcan was expressed in the articular surface, synovia, and cruciate ligaments at high levels (Fig 3D) in newborn *Prx1*-Cre/*Vcan*^+/+^ mice, whereas it was barely detected in *Prx1-Vcan* mice (Fig 3E). As the regions where both EGFP and Vcan are colocalized are likely affected in *Prx1-Vcan* mice, these observations implied that the abnormalities initiate in the ligament.

**Fig 4:**
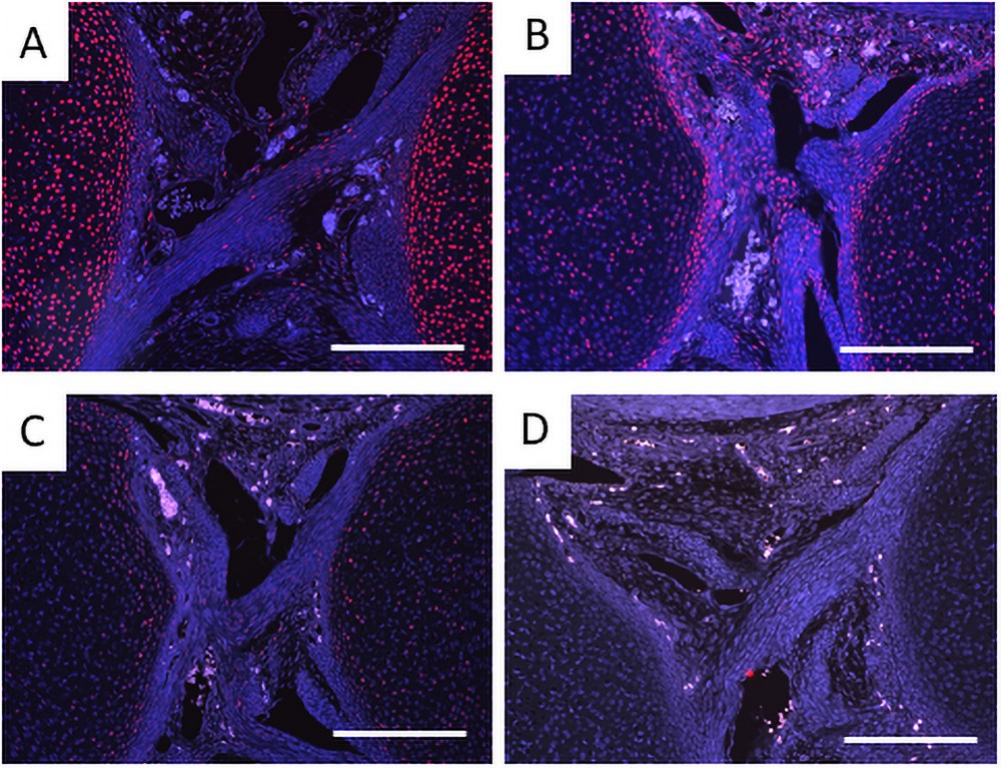
Scx immunostaining of newborn mouse knee joint. A. *Prx1-Vcan*^+/+^ mouse. Note that Scx is observed in articular chondrocytes and cells in the ligaments. B, C, D. *Prx1-Vcan* mouse. Note different immunostaining patterns. Scx is weakly immunostained at different levels (B, C, D) in articular chondrocytes (Bar = 200 μm).

As the cruciate ligaments appeared as the initial site of abnormalities, we hypothesized that the number or the localization of cells that form ligaments are altered in *Prx1-Vcan* mice. The cells that contribute to ligament formation express scleraxis (scx) [33]. Whereas scx was immunostained in most articular chondrocytes and cells in the ligaments of *Prx1*- *Vcan*^+/+^ mice (Fig 4A), it was immunostained only in some chondrocytes and ligamentocytes in *Prx1-Vcan* joints (Fig 4B–4D).

**Fig 5:**
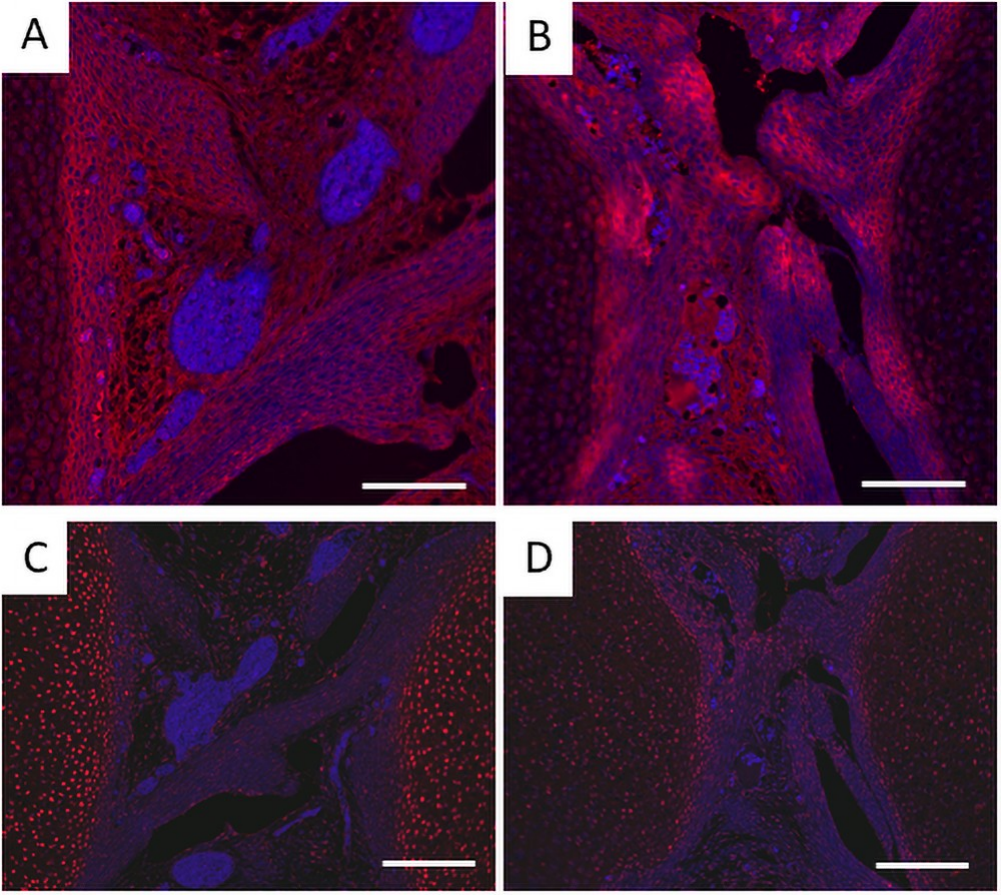
Immunostaining for type I and type II collagens of *Prx1-Vcan*^+/+^ (A, C) and *Prx1-Vcan* (B, D) mice. A, B. Whereas type I collagen is diffusely immunostained in the ligament of *Prx1-Vcan*^+/+^ (A), it is observed in spotty patterns in *Prx1-Vcan* mouse (B). Type I collagen is stained red, and the nuclei are counterstained blue. C, D. Whereas type II collagen is localized in articular chondrocytes of *Prx1-Vcan*^+/+^ joint (C), it is immunostained in some cells in the ligament of *Prx1-Vcan* joint (D) Type II collagen is stained red, and the nuclei are counterstained blue (Bar = 100 μm).

Progenitor cells of cruciate ligaments are localized in the joint interzone, and TGFβ shifts the balance of the cells from chondrogenesis to fibrogenesis [34]. Whereas type I collagen was immunostained associated with fibers in the *Prx1*-*Vcan*^+/+^ ligament, it was immunostained with spotty patterns in the *Prx1-Vcan* ligament (Fig 5A and 5B). Some cells were immunostained with type II collagen in *Prx1-Vcan* ligament, whereas type II collagen was barely detected in *Prx1*-*Vcan*^+/+^ ligament (Fig 5C and 5D). These results suggest that the *Prx1-Vcan* ligament contains cells with a chondrocytic phenotype.

## Discussion

In this study, we have demonstrated that Vcan expression in the joint of the perinatal period is required for normal development of the jointed appendages, especially of the cruciate ligaments. Although Vcan expression is limited to embryonic and perinatal periods in mice, its absence has profound effects on the joint function in the later ages. Immunohistochemical analysis of scx and collagens strongly suggests that Vcan contributes to the differentiation of the progenitor cells toward ligamentocytes and organization of type I collagen fibers of the ligaments.

We reported that *Prx1-Vcan* mice reveal impaired joint formation during development, which was likely limited to digits [28]. Here, our further investigation has revealed abnormalities in large joints, which become grossly apparent at five~six months. Our histological analysis has detected the disorganization of both ACL and PCL already in newborn joints. This is probably the cause of instability of the joint, leading to its destruction in later ages. In contrast, we found no apparent abnormalities in bone, joint capsules, and surrounding connective tissues. These results indicate that Vcan expressed in the jointed appendages generally plays an important role in joint formation and maintenance.

In the tendon and ligament cell lineage, scx, a transcription factor, is persistently expressed throughout differentiation and is known as a marker for the lineage [34]. Scx is observed in the primordia of the rotator cuff in the shoulder joint of E13.5 embryos and the collateral and anterior cruciate ligaments of the knee joints at E16.5 [33]. Analysis of double knockout mice of Scx and Sox9 has revealed that the Scx+ cell population can be subdivided into two distinct subpopulations of Scx+/Sox9+ and Scx+/Sox9– cells and that Sox9 expression later disappears from tendons and ligaments as differentiation proceeds [35]. These cells give rise to Scx–/Sox9+ chondrocytes and Scx+/Sox9– tenocytes/ligamentocytes. We have found a decrease in Scx+ chondrocytes in *Prx1-Vcan* joint cartilage, suggesting that it may lead to the impaired or delayed formation of tendons, ligaments, and cartilage. This is in agreement with the malformation of the ACL and PCL, and delayed cartilage development [28].

Vcan facilitates TGFβ-signaling by increasing the local concentration of TGFβ. TGFβ has dual effects on the development of cartilage and jointed appendages. Whereas TGFβ promotes chondrocyte differentiation in the early stages, it shifts the differentiation from chondrogenesis to fibrogenesis [36]. The local absence of Vcan may attenuate TGFβ-signaling, leading to a smaller progenitor population and less differentiation toward ligamentocytes. Whereas type I collagen was diffuse in the ligaments of *Prx1-Vcan*^+/+^ joints, it was immunostained in spotty patterns in those of *Prx1-Vcan* joints. Interestingly, type II collagen was immunostained in the ligaments of *Prx1-Vcan* joints, whereas it was not in the ligaments of *Prx1-Vcan*^+/+^ joints. The cruciate ligaments develop from cells that previously expressed *Col2a1*, which disappears in the joint interzone [37]. Our observations suggest that the chondrocytic cells expressing type II collagen remain in the ligaments of *Prx1-Vcan* joints, which may interfere with the organization of type I collagen fibers. Besides, local depletion of Vcan may affect them, as Vcan contributes to collagen fiber formation in developing dermis [38] and cancer stroma [39].

Vcan is expressed in ACL and the extra-articular medial collateral ligament in the normal adult canine joint [30]. Vcan is present among collagen fibrils and fibers, serving as a lubricant [40]. Although we could detect Vcan expression only in newborn joints, mechanical loading may induce Vcan expression in adult mice.

In humans, congenital absence of cruciate ligaments shows similar defects, and the patients acquire osteoarthritis at later ages [41, 42]. As Vcan complete knockout mice are embryonic lethal due to cardiac defects, Vcan is unlikely to be associated with this disease. Our study suggested that local TGFβ signaling is required for differentiation of Scx+/Sox9+ cells toward Scx+/Sox9- ligamentocytes. This congenital disorder may be associated with this process.

## Supporting information

S1 Fig Pictures obtained by microCT analysis of *Prx1-Vcan* and *Prx1-Vcan+/+* left joi nt. Analysis was performed for four mice, and the representative pictures are shown.
(TIF)

S2 Fig FACS analysis of dermal fibroblasts. Dermal fibroblasts were isolated and cultured for two passages and applied to FACS analysis. *Prx1-mT/mG* fibroblasts (A) and *mT/mG* fibroblasts (B) show similar patterns, indicating that most cells lack or had lacked *Prx1* promoter activity.
(PDF)

## References

[1] Islam S, Watanabe H. Versican: A Dynamic Regulator of the Extracellular Matrix. J Histochem Cytochem. 2020;68(11):763–75. Epub 2020/11/03. 10.1369/0022155420953922 . 33131383 PMC7649968

[2] Snow HE, Riccio LM, Mjaatvedt CH, Hoffman S, Capehart AA. Versican expression during skeletal/joint morphogenesis and patterning of muscle and nerve in the embryonic mouse limb. The anatomical recordPart A, Discoveries in molecular, cellular, and evolutionary biology. 2005;282(2):95–105. 10.1002/ar.a.20151 15633171

[3] Shepard JB, Krug HA, LaFoon BA, Hoffman S, Capehart AA. Versican expression during synovial joint morphogenesis. Int J Biol Sci. 2007;3(6):380–4. Epub 2007/09/13. 10.7150/ijbs.3.380 . 17848983 PMC1975773

[4] Matsumoto K, Kamiya N, Suwan K, Atsumi F, Shimizu K, Shinomura T, et al. Identification and characterization of versican/PG-M aggregates in cartilage. J Biol Chem. 2006;281(26):18257–63. Epub 2006/05/02. 10.1074/jbc.M510330200 . 16648631

[5] Isogai Z, Aspberg A, Keene DR, Ono RN, Reinhardt DP, Sakai LY. Versican interacts with fibrillin-1 and links extracellular microfibrils to other connective tissue networks. J Biol Chem. 2002;277(6):4565–72. Epub 2001/12/01. 10.1074/jbc.M110583200 . 11726670

[6] Aspberg A, Miura R, Bourdoulous S, Shimonaka M, Heinegard D, Schachner M, et al. The C-type lectin domains of lecticans, a family of aggregating chondroitin sulfate proteoglycans, bind tenascin-R by protein-protein interactions independent of carbohydrate moiety. Proceedings of the National Academy of Sciences of the United States of America. 1997;94(19):10116–21. 10.1073/pnas.94.19.10116 9294172 PMC23322

[7] Olin AI, Morgelin M, Sasaki T, Timpl R, Heinegard D, Aspberg A. The proteoglycans aggrecan and Versican form networks with fibulin-2 through their lectin domain binding. J Biol Chem. 2001;276(2):1253–61. Epub 2000/10/20. 10.1074/jbc.M006783200 . 11038354

[8] Day JM, Olin AI, Murdoch AD, Canfield A, Sasaki T, Timpl R, et al. Alternative splicing in the aggrecan G3 domain influences binding interactions with tenascin-C and other extracellular matrix proteins. J Biol Chem. 2004;279(13):12511–8. Epub 2004/01/15. 10.1074/jbc.M400242200 . 14722076

[9] Ujita M, Shinomura T, Ito K, Kitagawa Y, Kimata K. Expression and binding activity of the carboxyl-terminal portion of the core protein of PG-M, a large chondroitin sulfate proteoglycan. The Journal of biological chemistry. 1994;269(44):27603–9. 7961677

[10] Shinomura T, Nishida Y, Ito K, Kimata K. cDNA cloning of PG-M, a large chondroitin sulfate proteoglycan expressed during chondrogenesis in chick limb buds. Alternative spliced multiforms of PG-M and their relationships to versican. The Journal of biological chemistry. 1993;268(19):14461–9. 8314802

[11] Zimmermann DR, Dours-Zimmermann MT, Schubert M, Bruckner-Tuderman L. Versican is expressed in the proliferating zone in the epidermis and in association with the elastic network of the dermis. J Cell Biol. 1994;124(5):817–25. Epub 1994/03/01. 10.1083/jcb.124.5.817 . 8120102 PMC2119961

[12] Ito K, Shinomura T, Zako M, Ujita M, Kimata K. Multiple forms of mouse PG-M, a large chondroitin sulfate proteoglycan generated by alternative splicing. The Journal of biological chemistry. 1995;270(2):958–65. 10.1074/jbc.270.2.958 7822336

[13] Zako M, Shinomura T, Ujita M, Ito K, Kimata K. Expression of PG-M(V3), an alternatively spliced form of PG-M without a chondroitin sulfate attachment in region in mouse and human tissues. The Journal of biological chemistry. 1995;270(8):3914–8. 10.1074/jbc.270.8.3914 7876137

[14] Wu YJ, La Pierre DP, Wu J, Yee AJ, Yang BB. The interaction of versican with its binding partners. Cell research. 2005;15(7):483–94. 10.1038/sj.cr.7290318 16045811

[15] Yamagata M, Saga S, Kato M, Bernfield M, Kimata K. Selective distributions of proteoglycans and their ligands in pericellular matrix of cultured fibroblasts. Implications for their roles in cell-substratum adhesion. Journal of cell science. 1993;106 (Pt 1)(Pt 1):55–65. 8270643 10.1242/jcs.106.1.55

[16] Landolt RM, Vaughan L, Winterhalter KH, Zimmermann DR. Versican is selectively expressed in embryonic tissues that act as barriers to neural crest cell migration and axon outgrowth. Development (Cambridge, England). 1995;121(8):2303–12. 7671797 10.1242/dev.121.8.2303

[17] Bode-Lesniewska B, Dours-Zimmermann MT, Odermatt BF, Briner J, Heitz PU, Zimmermann DR. Distribution of the large aggregating proteoglycan versican in adult human tissues. The journal of histochemistry and cytochemistry: official journal of the Histochemistry Society. 1996;44(4):303–12. 10.1177/44.4.8601689 8601689

[18] Schmalfeldt M, Dours-Zimmermann MT, Winterhalter KH, Zimmermann DR. Versican V2 is a major extracellular matrix component of the mature bovine brain. The Journal of biological chemistry. 1998;273(25):15758–64. 10.1074/jbc.273.25.15758 9624174

[19] Henderson DJ, Copp AJ. Versican expression is associated with chamber specification, septation, and valvulogenesis in the developing mouse heart. Circulation research. 1998;83(5):523–32. 10.1161/01.res.83.5.523 9734475

[20] Kern CB, Norris RA, Thompson RP, Argraves WS, Fairey SE, Reyes L, et al. Versican proteolysis mediates myocardial regression during outflow tract development. Developmental dynamics: an official publication of the American Association of Anatomists. 2007;236(3):671–83. 10.1002/dvdy.21059 17226818 PMC1828600

[21] Kishimoto J, Ehama R, Wu L, Jiang S, Jiang N, Burgeson RE. Selective activation of the versican promoter by epithelial- mesenchymal interactions during hair follicle development. Proceedings of the National Academy of Sciences of the United States of America. 1999;96(13):7336–41. 10.1073/pnas.96.13.7336 10377415 PMC22086

[22] Soma T, Tajima M, Kishimoto J. Hair cycle-specific expression of versican in human hair follicles. J Dermatol Sci. 2005;39(3):147–54. Epub 2005/05/06. 10.1016/j.jdermsci.2005.03.010 . 15871917

[23] Sone S, Nakamura M, Maruya Y, Takahashi I, Mizoguchi I, Mayanagi H, et al. Expression of versican and ADAMTS during rat tooth eruption. J Mol Histol. 2005;36(4):281–8. Epub 2005/10/04. 10.1007/s10735-005-5534-2 . 16200461

[24] Yamagata M, Sanes JR. Versican in the developing brain: lamina-specific expression in interneuronal subsets and role in presynaptic maturation. J Neurosci. 2005;25(37):8457–67. Epub 2005/09/16. 10.1523/JNEUROSCI.1976-05.2005 . 16162928 PMC6725682

[25] Mjaatvedt CH, Yamamura H, Capehart AA, Turner D, Markwald RR. The Cspg2 gene, disrupted in the hdf mutant, is required for right cardiac chamber and endocardial cushion formation. Dev Biol. 1998;202(1):56–66. Epub 1998/10/06. 10.1006/dbio.1998.9001 . 9758703

[26] Hatano S, Kimata K, Hiraiwa N, Kusakabe M, Isogai Z, Adachi E, et al. Versican/PG-M is essential for ventricular septal formation subsequent to cardiac atrioventricular cushion development. Glycobiology. 2012;22(9):1268–77. Epub 2012/06/14. 10.1093/glycob/cws095 . 22692047

[27] Nandadasa S, Foulcer S, Apte SS. The multiple, complex roles of versican and its proteolytic turnover by ADAMTS proteases during embryogenesis. Matrix Biol. 2014;35:34–41. Epub 2014/01/22. 10.1016/j.matbio.2014.01.005 . 24444773 PMC5525047

[28] Choocheep K, Hatano S, Takagi H, Watanabe H, Kimata K, Kongtawelert P, et al. Versican facilitates chondrocyte differentiation and regulates joint morphogenesis. The Journal of biological chemistry. 2010;285(27):21114–25. 10.1074/jbc.M109.096479 20404343 PMC2898371

[29] Waggett AD, Ralphs JR, Kwan AP, Woodnutt D, Benjamin M. Characterization of collagens and proteoglycans at the insertion of the human Achilles tendon. Matrix Biol. 1998;16(8):457–70. Epub 1998/04/29. 10.1016/s0945-053x(98)90017-8 . 9550263

[30] Kharaz YA, Canty-Laird EG, Tew SR, Comerford EJ. Variations in internal structure, composition and protein distribution between intra- and extra-articular knee ligaments and tendons. J Anat. 2018;232(6):943–55. Epub 2018/03/03. 10.1111/joa.12802 . 29498035 PMC5978954

[31] Samiric T, Parkinson J, Ilic MZ, Cook J, Feller JA, Handley CJ. Changes in the composition of the extracellular matrix in patellar tendinopathy. Matrix Biol. 2009;28(4):230–6. Epub 2009/04/18. 10.1016/j.matbio.2009.04.001 . 19371780

[32] Jelinsky SA, Rodeo SA, Li J, Gulotta LV, Archambault JM, Seeherman HJ. Regulation of gene expression in human tendinopathy. BMC Musculoskelet Disord. 2011;12:86. Epub 2011/05/05. 10.1186/1471-2474-12-86 . 21539748 PMC3095578

[33] Asou Y, Nifuji A, Tsuji K, Shinomiya K, Olson EN, Koopman P, et al. Coordinated expression of scleraxis and Sox9 genes during embryonic development of tendons and cartilage. Journal of orthopaedic research: official publication of the Orthopaedic Research Society. 2002;20(4):827–33. 10.1016/S0736-0266(01)00169-3 12168674

[34] Schweitzer R, Chyung JH, Murtaugh LC, Brent AE, Rosen V, Olson EN, et al. Analysis of the tendon cell fate using Scleraxis, a specific marker for tendons and ligaments. Development (Cambridge, England). 2001;128(19):3855–66. 11585810 10.1242/dev.128.19.3855

[35] Sugimoto Y, Takimoto A, Akiyama H, Kist R, Scherer G, Nakamura T, et al. Scx+/Sox9+ progenitors contribute to the establishment of the junction between cartilage and tendon/ligament. Development (Cambridge, England). 2013;140(11):2280–8. 10.1242/dev.096354 23615282

[36] Lorda-Diez CI, Montero JA, Martinez-Cue C, Garcia-Porrero JA, Hurle JM. Transforming growth factors beta coordinate cartilage and tendon differentiation in the developing limb mesenchyme. The Journal of biological chemistry. 2009;284(43):29988–96. 10.1074/jbc.M109.014811 19717568 PMC2785627

[37] Hyde G, Boot-Handford RP, Wallis GA. Col2a1 lineage tracing reveals that the meniscus of the knee joint has a complex cellular origin. Journal of anatomy. 2008;213(5):531–8. 10.1111/j.1469-7580.2008.00966.x 19014360 PMC2667547

[38] Hatano S, Nagai N, Sugiura N, Tsuchimoto J, Isogai Z, Kimata K, et al. Versican A-subdomain is required for its adequate function in dermal development. Connect Tissue Res. 2018;59(2):178–90. Epub 2017/05/11. 10.1080/03008207.2017.1324432 . 28488903

[39] Fanhchaksai K, Okada F, Nagai N, Pothacharoen P, Kongtawelert P, Hatano S, et al. Host stromal versican is essential for cancer-associated fibroblast function to inhibit cancer growth. Int J Cancer. 2016;138(3):630–41. Epub 2015/08/14. 10.1002/ijc.29804 . 26270355

[40] Rumian AP, Wallace AL, Birch HL. Tendons and ligaments are anatomically distinct but overlap in molecular and morphological features—a comparative study in an ovine model. J Orthop Res. 2007;25(4):458–64. Epub 2007/01/06. 10.1002/jor.20218 . 17205554

[41] Berruto M, Gala L, Usellini E, Duci D, Marelli B. Congenital absence of the cruciate ligaments. Knee surgery, sports traumatology, arthroscopy: official journal of the ESSKA. 2012;20(8):1622–5. 10.1007/s00167-011-1816-2 22167203

[42] Lu R, Zhu DP, Chen N, Sun H, Li ZH, Cao XW. How should congenital absence of cruciate ligaments be treated? A case report and literature review. World journal of clinical cases. 2019;7(19):3082–9. 10.12998/wjcc.v7.i19.3082 31624758 PMC6795736

