# Supplementary figures and images for: Versican contributes to ligament formation of knee joints
Source: PLoS One. 2021 Apr 22;16(4):e0250366. doi: 10.1371/journal.pone.0250366 (PMC8061984; doi:10.1371/journal.pone.0250366)

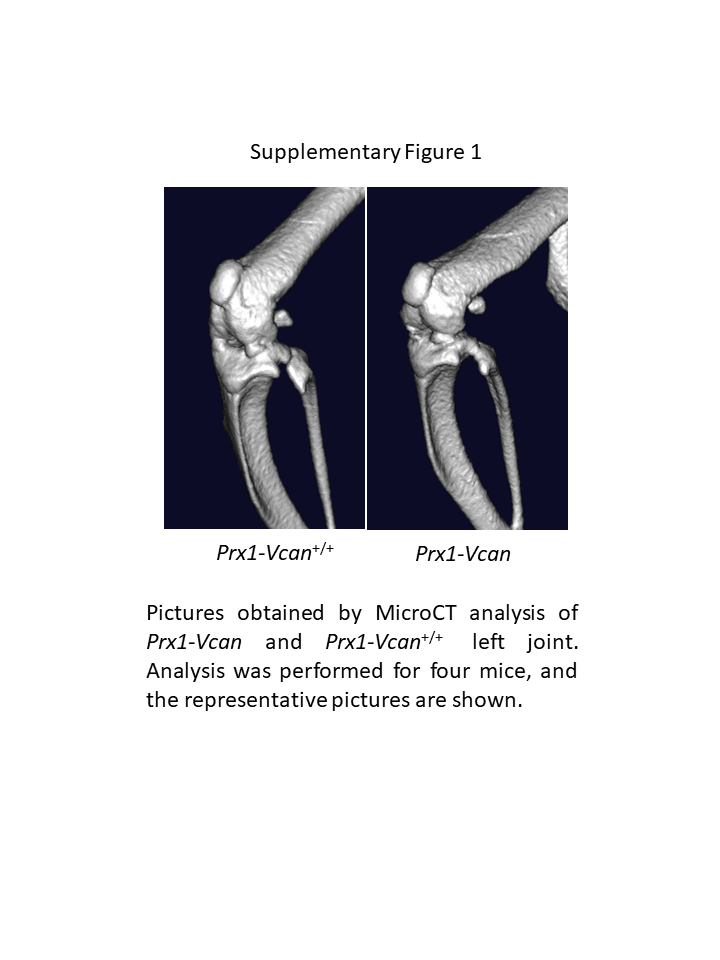

Supplement: S1 Fig — Analysis was performed for four mice, and the representative pictures are shown. (TIF) [file pone.0250366.s001.TIF]
