# Supplementary material for: Versican contributes to ligament formation of knee joints
Source: PLoS One. 2021 Apr 22;16(4):e0250366. doi: 10.1371/journal.pone.0250366 (PMC8061984; doi:10.1371/journal.pone.0250366)
Supplement: S2 Fig — Dermal fibroblasts were isolated and cultured for two passages and applied to FACS analysis. Prx1-mT/mG fibroblasts (A) and mT/mG fibroblasts (B) show similar patterns, indicating that most cells lack or had lacked Prx1 promoter activity. (PDF) [file pone.0250366.s002.pdf]

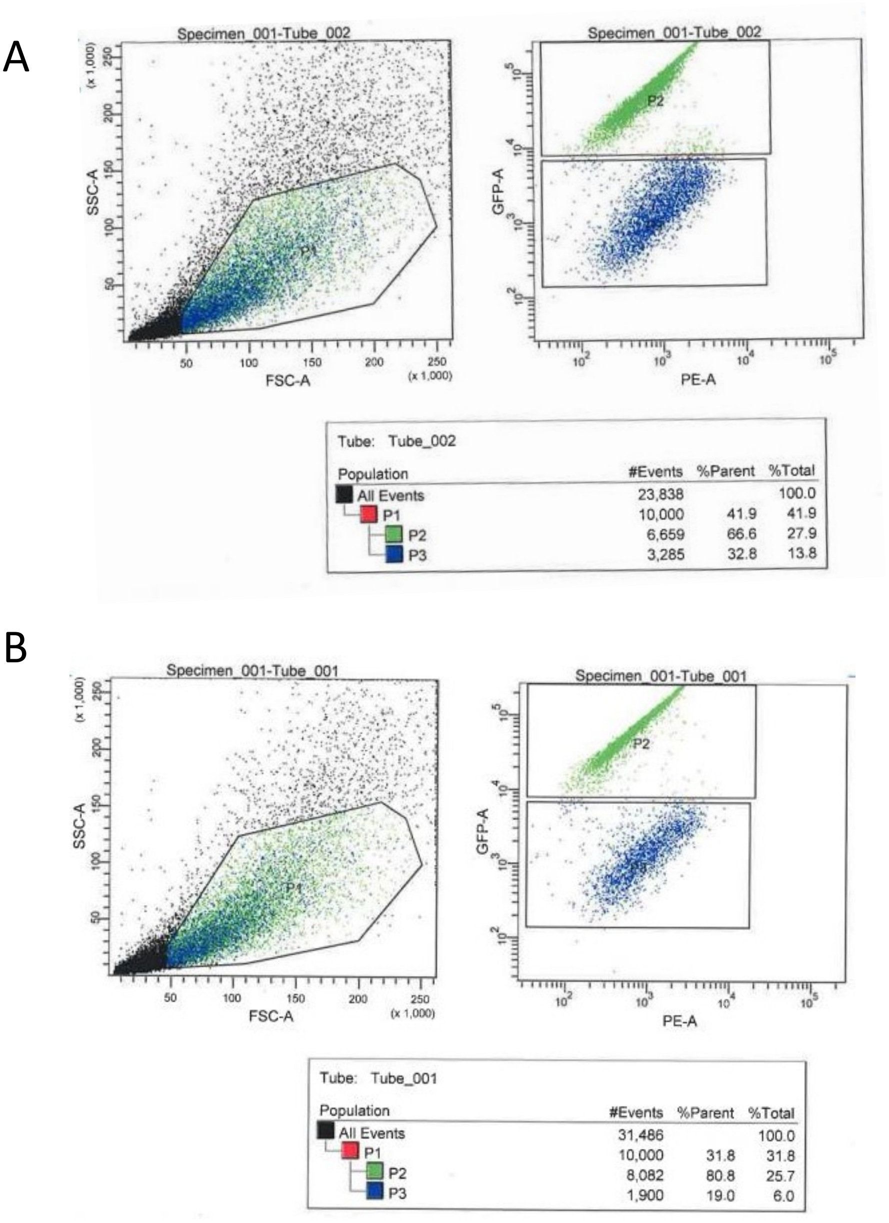

Population	#Events	%Parent	%Total
All Events	23,838		100.0
P1	10,000	41.9	41.9
P2	6,659	66.6	27.9
P3	3,285	32.8	13.8

Population	#Events	%Parent	%Total
All Events	31,486		100.0
P1	10,000	31.8	31.8
P2	8,082	80.8	25.7
P3	1,900	19.0	6.0

S2 Fig. FACS analysis of dermal fibroblasts. Dermal fibroblasts were isolated and cultured for two passages and applied to FACS analysis. *Prx1-mT/mG* fibroblasts (A) and *mT/mG* fibroblasts (B) show similar patterns, indicating that most cells lack or had lacked *Prx1* promoter activity.
